# Association of genetic variations in the lipid regulatory pathway genes FBXW7 and SREBPs with coronary artery disease among Han Chinese and Uygur Chinese populations in Xinjiang, China

**DOI:** 10.18632/oncotarget.21082

**Published:** 2017-09-19

**Authors:** Asiya Abudesimu, Dilare Adi, Dilixiati Siti, Xiang Xie, Yi-Ning Yang, Xiao-Mei Li, Ying-Hong Wang, Yong-Tao Wang, Ya-Jie Meng, Fen Liu, Bang-Dang Chen, Xiang Ma, Zhen-Yan Fu, Yi-Tong Ma

**Affiliations:** ^1^ Department of Cardiology, First Affiliated Hospital of Xinjiang Medical University, Urumqi, PR China; ^2^ Xinjiang Key Laboratory of Cardiovascular Disease, Clinical Medical Research Institute of First Affiliated Hospital of Xinjiang Medical University, Urumqi, PR China

**Keywords:** SREBP family, FBXW7, coronary artery disease, case-control study

## Abstract

**Background:**

Hyperlipidemia is a major risk factor for coronary artery disease (CAD). The current study was designed to explore the possible correlation between single nucleotide polymorphisms (SNPs) in the lipid homeostasis regulatory genes F-box and WD repeat domain–containing 7 (FBXW7) and sterol regulatory element-binding proteins (SREBPs) with CAD among Han Chinese and Uygur Chinese populations in Xinjiang, China.

**Results:**

In the Uygur Chinese population, rs9902941 in SREBP-1 and rs10033601 in FBXW7 were found to be associated with CAD in a recessive model (TT vs. CT + CC, *P* = 0.032; GG vs. AG + AA, *P* = 0.010, respectively), and rs7288536 in SREBP-2 was found to be associated with CAD in an additive model (CT vs. CC + TT, *P* = 0.045). The difference was statistically significant in the Uygur Chinese population after multivariate adjustments [Odds ratio (OR) = 1.803, 95% confidence interval (CI): 1.036~3.137, *P* = 0.037; OR = 1.628, 95% CI: 1.080~2.454, *P* = 0.020; OR = 1.368; and 95% CI: 1.018~1.837, *P* = 0.037, respectively]. There were also significant interactions between the above-mentioned models in the Uygur Chinese population. However, these relationships were not observed before or after multivariate adjustment in the Han Chinese population.

**Materials and Methods:**

A total of 1,312 Han Chinese (650 CAD patients and 662 controls) and 834 Uygur Chinese (414 CAD patients and 420 controls) were enrolled in this case-control study. Three SNPs (rs9902941 in SREBP-1, rs7288536 in SREBP-2 and rs10033601 in FBXW7) were selected and genotyped using the improved multiplex ligase detection reaction (iMLDR) method.

**Conclusions:**

The results of this study indicate that variations in the lipid regulatory pathway genes FBXW7 and SREBPs (rs9902941 in SREBP-1, rs7288536 in SREBP-2 and rs10033601 in FBXW7) are associated with CAD in the Uygur Chinese population in Xinjiang, China.

## INTRODUCTION

Coronary artery disease (CAD) is the most common chronic disease; it is becoming increasingly prevalent and remains the leading cause of disability and mortality throughout the world [1–3]. CAD is a multifactorial disease that results from both genetic and environmental risk factors. Hyperlipidemia is one of the major independent risk factors for the development of CAD; approximatively 50% of CAD cases are associated with hyperlipidemia [4, 5]. Accumulated evidence suggests that genetic factors account for 40%~60% of the variation in plasma lipid concentrations and components [6, 7].

Lipid metabolism is regulated by a family of transcription factors known as sterol regulatory element-binding proteins (SREBPs) [8]. The SREBP family consists of three members: SREBP-1a and 1c are produced from a single gene named SREBP-1 [9]; and SREBP-2 is encoded by a separate gene named SREBP-2 [10]. The SREBP family controls cholesterol and lipid synthesis by activating the expression of SREBP target genes, such as fatty acid synthase, 3-hydroxy-3-methylglutaryl-CoA (HMG-CoA) reductase, HMG-CoA synthase, and the low-density lipoprotein (LDL) receptor [11, 12]. Thus, the SREBPs are considered master regulators of cholesterogenesis and lipogenesis.

A previous study reported that mature SREBPs are highly unstable due to their susceptibility to ubiquitin-dependent degradation [13]. Nevertheless, ubiquitin-dependent degradation effectively maintains the balance of cholesterol and lipid levels in the nucleus. It has been shown that F-box and WD repeat domain–containing 7 (FBXW7) is a ubiquitin-E3 ligase-targeting factor that mediates the recognition of phosphorylated substrates, such as cyclin E, c-Myc, c-Jun and SREBPs, for proteolysis [14–18]. Considerable evidence from previous studies has indicated that FBXW7 controls the degradation of all members of the SREBP family [19, 20]. FBXW7 interacts with nuclear SREBP family genes and enhances their ubiquitination, which leads to their degradation. In contrast, inactivation of endogenous FBXW7 results in the stabilization of SREBP family genes, which then induces the expression of endogenous SREBP target genes and enhances the synthesis of cholesterol and fatty acids as well as the uptake of LDL [21]. Therefore, we hypothesized that the lipid regulatory pathway genes FBXW7 and SREBPs are associated with CAD. The identification of genetic alterations may lead to a novel understanding of CAD development. In addition, some genetic alterations within the lipid regulatory pathway may contribute to CAD susceptibility and serve as druggable targets for the disease. To date, no case-control studies have been conducted to assess the association of genetic variations in the lipid regulatory pathway genes FBXW7 and SREBPs with CAD in different ethnic groups.Thus, the current study was designed to explore the possible correlation between single nucleotide polymorphisms (SNPs) in the lipid regulatory pathway genes FBXW7 and SREBPs (rs9902941 in SREBP-1, rs7288536 in SREBP-2 and rs10033601 in FBXW7) with CAD among Han Chinese and Uygur Chinese populations in Xinjiang, China.

## RESULTS

### General characteristics of the study participants

The general characteristics of the Han Chinese population are listed in Table 1. A total of 650 patients with CAD and 662 healthy controls were enrolled in the present study. Among the CAD patients, 230 (35.4%) were women and 420 (64.6%) were men, and the mean age was 57.73 ± 7.80 years old. Among the controls, 237 (35.8%) were women and 425 (64.2%) were men, and the mean age was 58.36 ± 7.49 years old. There were significant differences in the following parameters between the CAD and control groups: smoking status (*P* = 0.004), drinking status (*P* = 0.001), hypertension (*P <* 0.001), diabetes (*P <* 0.001), hyperlipidemia (*P <* 0.001), fasting plasma glucose (FPG, *P <* 0.001), total cholesterol (TC, *P <* 0.001), high density lipoprotein cholesterol (HDL-C, *P* = 0.011) and low-density lipoprotein cholesterol (LDL-C, *P <* 0.001). However, we did not observe significant differences between patients and controls regarding age (*P* = 0.136), gender (*P* = 0.875) and body mass index (BMI, *P* = 0.110) or triglyceride (TG, *P* = 0.404), uric acid (*P* = 0.369), blood urea nitrogen (BUN, *P* = 0.168) and creatinine (Cr, *P* = 0.281) levels.

**Table 1:** General characteristics of the study participants (Han Chinese)

Characteristics	CAD (*n* = 650)	Control (*n* = 662)	χ2 or *t*	*P*
Age,(years)	57.73 ± 7.80	58.36 ± 7.49	1.493	0.136
Gender (male) (%)	420 (64.6%)	425 (64.2%)	0.025	0.875
BMI (kg/m^2^)	25.58 ± 3.14	25.31 ± 3.04	1.597	0.110
Smoking (%)	344 (52.9%)	297 (44.9%)	8.525	0.004
Drinking (%)	247 (38.0%)	195 (29.5%)	10.717	0.001
Hypertension (%)	396 (60.9%)	306 (46.2%)	28.487	< 0.001
Diabetes (%)	231 (35.5%)	164 (24.8%)	18.062	< 0.001
Hyperlipidemia (%)	222 (34.2%)	156 (23.6%)	17.929	< 0.001
FPG, (mmol/L)	6.43 ± 2.98	5.65 ± 2.07	5.502	< 0.001
TG, (mmol/L)	1.83 ± 1.15	1.78 ± 1.16	1.926	0.404
TC, (mmol/L)	4.01 ± 1.03	3.79 ± 1.13	3.787	< 0.001
HDL-C, (mmol/L)	1.06 ± 0.30	1.11 ± 0.33	2.531	0.011
LDL-C, (mmol/L)	2.76 ± 0.71	2.58 ± 0.87	4.041	< 0.001
Uric acid, (μmol/L)	314.98 ± 85.59	319.11 ± 80.35	0.899	0.369
BUN, (mmol/L)	5.40 ± 1.41	5.29 ± 1.55	1.380	0.168
Cr, (mmol/L)	73.40 ± 16.18	72.37 ± 18.29	1.079	0.281

BMI, body mass index; FPG, fasting plasma glucose; TG, triglyceride; TC, total cholesterol; HDL-C, high density lipoprotein cholesterol; LDL-C, low density lipoprotein cholesterol; BUN, blood urea nitrogen; Cr, creatinine.

The *P* value of the continuous variables was calculated by the independent-sample *t*-test. The *P* value of the categorical variables was calculated by χ2 test.

The general characteristics of the Uygur Chinese population are listed in Table 2. There were 414 patients with CAD and 420 healthy controls. Among the CAD patients, 98 (23.7%) were women and 316 (76.3%) were men, and the mean age was 58.00 ± 7.56 years old. Among the controls, 107 (25.5%) were women and 313 (74.5%) were men, and the mean age was 57.63 ± 7.48 years old. There were significant differences in the following parameters between the CAD and control groups: smoking status (*P* = 0.029), drinking status (*P* = 0.004), hypertension (*P <* 0.001), diabetes (*P <* 0.001), hyperlipidemia (*P <* 0.001) and FPG (*P <* 0.001) as well as TG (*P* = 0.007), TC (*P* = 0.029), HDL-C (*P <* 0.001), LDL-C (*P <* 0.001) and uric acid (*P* = 0.021) levels. However, we did not observe significant differences between patients and controls regarding age (*P* = 0.476), gender (*P* = 0.545), BMI (*P* = 0.794) and BUN (*P* = 0.716).

**Table 2:** General characteristics of the study participants (Uygur Chinese)

Characteristics	CAD (*n* = 414)	Control (*n* = 420)	χ2 or *t*	*P*
Age, (years)	58.00 ± 7.56	57.63 ± 7.48	0.713	0.476
Gender (male) (%)	316 (76.3%)	313 (74.5%)	0.366	0.545
BMI (kg/m^2^)	26.91 ± 3.34	26.85 ± 3.89	0.262	0.794
Smoking (%)	136 (32.9%)	109 (26.0%)	4.781	0.029
Drinking (%)	114 (27.5%)	80 (19.0%)	8.416	0.004
Hypertension (%)	250 (60.4%)	195 (46.4%)	16.321	< 0.001
Diabetes (%)	176 (42.5%)	99 (23.6%)	33.842	< 0.001
Hyperlipidemia (%)	330 (79.7%)	270 (64.3%)	24.573	< 0.001
FPG, (mmol/L)	6.55 ± 3.08	5.79 ± 2.45	3.904	< 0.001
TG, (mmol/L)	2.03 ± 1.19	1.79 ± 1.40	2.716	0.007
TC, (mmol/L)	4.15 ± 1.13	3.99 ± 0.91	2.184	0.029
HDL-C, (mmol/L)	0.90 ± 0.33	1.00 ± 0.33	4.448	< 0.001
LDL-C, (mmol/L)	2.88 ± 0.60	2.62 ± 0.43	7.091	< 0.001
Uric acid, (μmol/L)	315.12 ± 85.71	302.28 ± 73.95	2.315	0.021
BUN, (mmol/L)	5.52 ± 1.85	5.48 ± 1.76	0.364	0.716
Cr, (mmol/L)	75.92 ± 21.31	72.13 ± 15.95	2.907	0.004

BMI, body mass index; FPG, fasting plasma glucose; TG, triglyceride; TC, total cholesterol; HDL-C, high density lipoprotein cholesterol; LDL-C, low density lipoprotein cholesterol; BUN, blood urea nitrogen; Cr, creatinine.

The *P* value of the continuous variables was calculated by the independent-sample *t*-test. The *P* value of the categorical variables was calculated by χ2 test.

### The genotype distribution of selected SNPs in CAD patients and controls

Tables 3 and 4 show the genotype distributions of selected SNPs in patients with CAD and control participants. In the Han Chinese and Uygur Chinese populations, the genotype distributions of the three SNPs for both CAD patients and controls were in accordance with the Hardy-Weinberg equilibrium (data not shown).

**Table 3:** Genotypes distribution of the lipid regulatory pathway genes FBXW7 and SREBPs in Han Chinese

Variants	CAD *n*(%)	Control *n* (%)	*χ2*	*P*-value
SREBP-1 rs9902941 C < T				
CC	534 (82.2%)	549 (82.9%)	1.469	0.480
CT	111 (17.1%)	104 (15.7%)		
TT	5 (0.8%)	9 (1.4%)		
Dominant model				
CC	534 (82.2%)	549 (82.9%)	0.137	0.711
CT + TT	116 (17.8%)	113 (17.1%)		
Recessive model				
TT	5 (0.8%)	12 (1.8%)	2.792	0.095
CT + CC	645 (99.2%)	650 (98.2%)		
Additive model				
CT	111 (17.1%)	101 (15.3%)	0.802	0.370
CC + TT	539 (82.9%)	561 (84.7%)		
SREBP-2 rs7288536 C < T				
CC	78 (12.0%)	67 (10.1%)	1.223	0.542
CT	266 (40.9%)	273 (41.2%)		
TT	306 (47.1%)	322 (48.6%)		
Dominant model				
CC	306 (47.1%)	322 (48.6%)	0.321	0.571
CT + TT	344 (52.9%)	340 (51.4%)		
Recessive model				
TT	78 (12.0%)	67 (10.1%)	1.178	0.278
CT + CC	572 (88.0%)	595 (89.9%)		
Additive model				
CT	266 (40.9%)	273 (41.2%)	0.013	0.908
CC + TT	384 (59.1%)	389 (58.8%)		
FBXW7 rs10033601 A < G				
AA	225 (34.6%)	218 (32.9%)	0.459	0.795
AG	294 (45.2%)	310 (46.8%)		
GG	131 (20.2%)	134 (20.2%)		
Dominant model				
AA	225 (34.6%)	218 (32.9%)	0.416	0.519
AG + GG	425 (65.4%)	444 (67.1%)		
Recessive model				
GG	131 (20.2%)	134 (20.2%)	0.002	0.968
AG + AA	519 (79.8%)	528 (79.8%)		
Additive model				
AG	294 (45.2%)	310 (46.8%)	0.337	0.562
AA + GG	356 (54.8%)	352 (53.2%)		

χ^2^ test for genotype distributions between coronary artery disease patients and controls.

**Table 4:** Genotypes distribution of the lipid regulatory pathway genes FBXW7 and SREBPs in Uygur Chinese

Variants	CAD *n* (%)	Control *n* (%)	*χ2*	*P*-value
SREBP-1 rs9902941 C < T				
CC	220 (53.1%)	224 (53.3%)	4.987	0.083
CT	154 (37.2%)	172 (41.0%)		
TT	40 (9.7%)	24 (5.7%)		
Dominant model				
CC	220 (53.1%)	224 (53.3%)	0.003	0.955
CT + TT	194 (46.9%)	196 (46.7%)		
Recessive model				
TT	40 (9.7%)	24 (5.7%)	4.586	0.032
CT + CC	374 (90.3%)	396 (94.3%)		
Additive model				
CT	154 (37.2%)	172 (41.0%)	1.234	0.267
CC + TT	260 (62.8%)	248 (59.0%)		
SREBP-2 rs7288536 C < T				
CC	41 (9.9%)	49 (11.7%)	4.058	0.131
CT	189 (45.7%)	163 (38.8%)		
TT	184 (44.4%)	208 (49.5%)		
Dominant model				
CC	184 (44.4%)	208 (49.5%)	2.159	0.142
CT + TT	230 (55.6%)	212 (50.5%)		
Recessive model				
TT	41 (9.9%)	49 (11.7%)	0.673	0.412
CT + CC	373 (90.1%)	371 (88.3%)		
Additive model				
CT	189 (45.9%)	163 (38.8%)	4.002	0.045
CC + TT	225 (54.3%)	257 (61.2%)		
FBXW7 rs10033601 A < G				
AA	152 (36.7%)	163 (38.8%)	6.750	0.034
AG	185 (44.7%)	206 (49%)		
GG	77 (18.6%)	51 (12.1%)		
Dominant model				
AA	152 (36.7%)	163 (38.8%)	0.389	0.533
AG + GG	262 (63.3%)	257 (61.2%)		
Recessive model				
GG	77 (18.6%)	51 (12.1%)	6.689	0.010
AG + AA	337 (81.4%)	369 (87.9%)		
Additive model				
AG	185 (44.7%)	206 (49%)	1.593	0.207
AA + GG	229 (55.3%)	214 (51%)		

χ^2^ test for genotype distributions between coronary artery disease patients and controls.

In the Han Chinese population, there were no significant differences in the distribution of genotypes and models (dominant, recessive and additive) for variants in SREBP-1 (rs9902941), SREBP-2 (rs7288536) and FBXW7 (rs10033601) between the CAD and control groups (Table 3).

In the Uygur Chinese population, variant rs9902941 of SREBP-1 exhibited significant differences between the CAD and control groups in a recessive model (TT vs. CT + CC, *P* = 0.032), nevertheless, the difference was no longer significant after Bonferroni's correction (*P* > 0.05/3 = 0.0167). And there were no significant differences in the distribution of genotypes (*P* = 0.083), in a dominant model (CC vs. CT + TT, *P* = 0.955) or in an additive model (CT vs. CC + TT, *P* = 0.267). Variant rs7288536 of SREBP-2 exhibited significant differences between the two groups in an additive model (CT vs. CC + TT, *P* = 0.045), nevertheless, the difference was no longer significant after Bonferroni's correction (*P* > 0.05/3 = 0.0167). And there were no significant differences in the distribution of genotypes (*P* = 0.131), in a dominant model (CC vs. CT + TT, *P* = 0.142) or in a recessive model (TT vs. CT + CC, *P* = 0.412). Similarly, variant rs10033601 of FBXW7 exhibited significant differences between the two groups in the distribution of genotypes (*P* = 0.034) and in a recessive model (GG vs. AG + AA, *P* = 0.010), Nevertheless, the difference of the distribution of genotypes (*P* = 0.034) was no longer significant after Bonferroni's correction (*P* > 0.05/3 = 0.0167). And there were no significant differences in a dominant model (AA vs. AG + GG, *P* = 0.533) or an additive model (AG vs. AA + GG, *P* = 0.207) (Table 4).

### Multiple logistic regression analysis for CAD patients and control subjects in the Uygur Chinese population

As shown in Table 5, in the Uygur Chinese population, an association was observed in a recessive model for rs9902941 of SREBP-1 after adjusting for confounding factors of CAD, such as age; gender; plasma concentrations of TG, TC, HDL-C, LDL-C and FPG; hypertension; drinking; and smoking, using multivariate logistic regression analysis (OR = 1.803, 95% CI: 1.036~3.137, *P* = 0.037). Regarding rs7288536 of SREBP-2, a significant difference was observed in an additive model after multivariate adjustment (OR = 1.368, 95% CI: 1.018~ 1.837, *P* = 0.037). Similarly, for rs10033601 of FBXW7, an association was observed in a recessive model after multivariate adjustment (OR = 1.628, 95% CI: 1.080~2.454, *P* = 0.020).

**Table 5:** Multiple logistic regression analysis for CAD patients and control subjects in Uygur Chinese

Variants	Factors	B	S.E.	Wald	*P*	OR	95% CI
SREBP-1 rs9902941	Recessive model	0.589	0.283	4.347	0.037	1.803	1.036–3.137
	Age	0.016	0.011	2.224	0.136	1.016	0.995–1.039
	Gender	−0.129	0.192	0.449	0.503	0.879	0.603–1.281
	Smoking	0.228	0.200	1.294	0.255	1.256	0.848–1.859
	Drinking	0.475	0.204	5.423	0.020	1.608	1.078–2.400
	Hypertension	0.495	0.152	10.661	0.001	1.640	1.219–2.207
	FPG	0.077	0.029	7.249	0.007	1.080	1.021–1.143
	TG	0.062	0.060	1.046	0.307	1.064	0.945–1.198
	TC	−0.054	0.088	0.378	0.539	0.947	0.797–1.126
	HDL-C	−0.913	0.263	12.085	0.001	0.401	0.240–0.671
	LDL-C	1.017	0.166	37.687	< 0.001	2.765	1.998–3.825
SREBP-2 rs7288536	Additive model	0.313	0.151	4.331	0.037	1.368	1.018–1.837
	Age	0.017	0.011	2.527	0.112	1.018	0.996–1.040
	Gender	−0.116	0.192	0.365	0.546	0.890	0.611–1.298
	Smoking	0.248	0.200	1.545	0.214	1.282	0.866–1.897
	Drinking	0.517	0.204	6.418	0.011	1.677	1.124–2.503
	Hypertension	0.487	0.151	10.343	0.001	1.628	1.210–2.190
	FPG	0.078	0.029	7.410	0.006	1.081	1.022–1.144
	TG	0.059	0.061	0.934	0.334	1.061	0.941–1.195
	TC	−0.050	0.089	0.316	0.574	0.951	0.799–1.133
	HDL-C	−0.883	0.268	10.877	0.001	0.414	0.245–0.699
	LDL-C	1.018	0.166	37.785	< 0.001	2.767	2.000–3.828
FBXW7 rs10033601	Recessive model	0.487	0.209	5.413	0.020	1.628	1.080–2.454
	Age	0.017	0.011	2.539	0.111	1.018	0.996–1.040
	Gender	−0.151	0.193	0.612	0.434	0.860	0.590–1.255
	Smoking	0.251	0.200	1.575	0.210	1.285	0.868–1.902
	Drinking	0.512	0.204	6.305	0.012	1.669	1.119–2.490
	Hypertension	0.476	0.152	9.861	0.002	1.610	1.196–2.166
	FPG	0.082	0.029	8.143	0.004	1.086	1.026–1.149
	TG	0.061	0.061	0.994	0.319	1.062	0.943–1.197
	TC	−0.065	0.089	0.522	0.470	0.937	0.787–1.117
	HDL-C	−0.892	0.266	11.227	0.001	0.410	0.243–0.691
	LDL-C	1.012	0.166	37.327	< 0.001	2.752	1.989–3.807

FPG, fasting plasma glucose; TG, triglyceride; TC, total cholesterol; HDL-C, high density lipoprotein cholesterol; LDL-C, low density lipoprotein cholesterol

### The genotype distribution of selected SNPs in CAD patients with and without diabetes

In the Uygur Chinese population, the genotype distributions of the three SNPs among both CAD patients with and without diabetes were in accordance with the Hardy-Weinberg equilibrium (data not shown).

To avoid diabetes as a confounder for our association study, we further investigated whether the association of the SNPs with CAD was due to an association with diabetes. Thus, we tested the association of the SNPs with diabetes in CAD subjects and revealed that there were no significant differences in the distribution of genotypes and models (dominant, recessive and additive) for variants in SREBP-1 (rs9902941), SREBP-2 (rs7288536) and FBXW7 (rs10033601) between the CAD patients with diabetes and CAD patients without diabetes (Supplementary Table 1).

### Gene-gene interactions in Uygur Chinese using multiple logistic regression analysis

We further investigated the gene-gene interactions in the Uygur Chinese population and found a significant interaction between the recessive model (TT vs. CT+CC) of SREBP-1 and the additive model (CT vs. CC + TT) of SREBP-2 (Table 6). This interaction was associated with an increased risk of CAD (OR = 1.396, 95% CI: 1.120–1.740, *P* = 0.003). There was also an interaction between a recessive model of SREBP-1 and a recessive model (GG vs. AG + AA) of FBXW7. Moreover, this interaction significantly increased CAD risk (OR = 1.710, 95% CI: 1.225–2.388, *P* = 0.002). There was also a significant interaction between an additive model of SREBP-2 and a recessive model of FBXW7 in CAD risk (OR = 1.327, 95% CI: 1.094–1.611, *P* = 0.004). Finally, the combination of these three models exhibited a significant interaction that was associated with an increased risk of CAD (OR = 1.342, 95% CI = 1.137–1.584, *P* = 0.001).

**Table 6:** Multiple logistic regression analysis of the gene-gene interaction in Uygur Chinese

Factors	*B*	*S.E.*	*Wald*	*P*	*95 % CI*	*OR*
SREBP-1_(recessive)_× SREBP-2_(additive)_	0.334	0.112	8.820	0.003	1.120–1.740	1.396
SREBP-1_(recessive)_× FBXW7_(recessive)_	0.537	0.170	9.943	0.002	1.225–2.388	1.710
SREBP-2_(additive)_ × FBXW7_(recessive)_	0.283	0.099	8.215	0.004	1.094–1.611	1.327
SREBP-1_(recessive)_× SREBP-2_(additive)_× FBXW7_(recessive)_	0.294	0.085	12.087	0.001	1.137–1.584	1.342

SREBP-1_(recessive),_ TT vs. CT+CC; SREBP-2_(additive),_ CT vs. CC + TT; FBXW7_(recessive),_ GG vs. AG + AA Adjusted for age, gender, smoking, drinking, hypertension, FPG, TG, TC, HDL-C, LDL-C.

## DISCUSSION

In the present case-control study, we found that variations rs9902941 of SREBP-1, rs7288536 of SREBP-2 and rs10033601 of FBXW7 were associated with CAD in the Uygur Chinese population but were not associated with CAD in the Han Chinese population. To the best of our knowledge, this was the first study to investigate the association of genetic variations in the lipid regulatory pathway genes FBXW7 and SREBP with CAD in the Uygur Chinese population.

Disorders of lipid metabolism are involved in the pathogenesis of CAD [22, 23]. SREBP family genes are canonical lipid regulatory pathway genes involved in the homeostasis of serum lipids. To date, accumulated evidence generated from different study groups has suggested that genetic polymorphisms of the SREBP family genes are associated with major risk factors for CAD, such as dyslipidemia, high FPG and BMI [24–26]. The polymorphism −36delG in the 5′untranslated region of the SREBP-1 gene has been shown to be associated with atherogenic lipid profiles and the development of atherosclerosis [27]. Laaksonen R et al. also found that polymorphism rs2297508 was associated with an atherogenic lipid profile [28]. In addition, Rios et al. showed that an interaction between the −36delG polymorphism in SREBP-1 and apolipoprotein B (ApoB) polymorphisms influences the TC and LDL levels in patients with CAD from a Brazilian population of European descent [24]. Another study conducted in the Caucasian population indicated an association of the rs11868035 polymorphism in SREBP-1 with increased TC and LDL-C levels [26]. In addition, a previous study conducted by Chien KL, et al. reported that a strong association of the SREBP-1 rs9902941 polymorphism with the reduction of LDL-C after statin treatment, and it was observed among Chinese patients with hypercholesterolemia [29]. In our study we observed that the distribution of the recessive model (TT vs. CT+CC) of SREBP-1 rs9902941 was significantly higher among CAD patients compared to control subjects. The difference remained significant after adjusting for confounding factors of CAD, such as age; gender; plasma concentrations of TG, TC, HDL-C, LDL-C and FPG; hypertension; drinking and smoking. The results indicated that individuals with the TT genotype of rs9902941 may be at increased risk for CAD.

Regarding the SREBP-2 gene, there was a study that indicated an association between serum lipid level and the G595A polymorphism in the SREBP-2 gene [30, 31]. Furthermore, the G595A polymorphism of SREBP-2 has also been shown to be associated with intima media thickness in asymptomatic hypercholesterolemic men [32]. Another study provided evidence for the association between the SNPs rs1052717 and rs2267443 in SREBP-2 and metabolic syndrome in schizophrenic patients treated with clozapine [33]. In addition, the functional SNP rs133291 C/T in the SREBP-2 gene has been linked to serum LDL-C [34]. An additional study provided evidence that the rs2228314 G>C polymorphism in SREBP-2 may contribute to sudden cardiac death in early middle-aged men [35]. However, Chen Z et al. assessed the association of rs2228314 and rs12487736 with premature CAD in a relatively young cohort from Eastern China and did not observe any associations of rs2228314 and rs12487736 with premature CAD. In our report, we found that rs7288536, another SNP in the SREBP-2 gene, is associated with CAD in the Uygur population from Western China. Our results indicated that the distribution of the additive model (CT vs. CC + TT) of SREBP-2 rs7288536 was significantly higher among CAD patients than control subjects. The difference remained significant after adjusting for confounding factors of CAD. These findings suggest that individuals with the CT genotype of rs7288536 may be at increased risk for CAD

Our results might due to some mechanism which modify the function of SREBPs, then promotes fatty acid synthesis and lipogenesis, thereby contributing to the development of atherosclerosis [24]. In addition, overexpression of SREBPs could also be involved in obesity [26]. All of these effects may promote the development and progression of CAD. Moreover, the overexpression of SREBPs could be a factor responsible for insulin resistance through the overaccumulation of lipids [36]. Therefore, to avoid diabetes as a confounder for our association study, we further investigated whether the association of the SNPs with CAD was due to an association with diabetes, and the results showed that there was no association between the SNPs and diabetes. However, the exact underlying mechanism by which these polymorphisms confer CAD susceptibility remains to be elucidated.

As a major regulator of lipid metabolism, FBXW7 degrades the SREBP family by controlling the phosphorylation of T426 and S430 via GSK-3 [19]. A reliably study by Onoyama I et al. showed that FBXW7 regulates lipid metabolism and cell fate decisions in the mouse liver [20]. In addition, another study showed that FBXW7 controls adipocyte differentiation by targeting C/EBPα for degradation, and thus, FBXW7 could be an important regulator of energy and lipid metabolism [37]. Therefore, we hypothesized that the FBXW7 gene might be associated with CAD. However, the relationship between the FBXW7 gene and cardiovascular diseases has not yet been studied. In our study, we observed that the distribution of the recessive model (GG vs. AG + AA) of FBXW7 rs10033601 was significantly higher among CAD patients than control subjects. The difference remained statistically significant after adjusting for the confounding factors of CAD. These findings suggest individuals with the GG genotype of rs10033601 may be at increased risk for CAD. The potential mechanism for this association is not yet clear, but we hypothesize it probably involves the inactivation of endogenous FBXW7, thus resulting in the stabilization of SREBP family genes. This would promote the expression of endogenous SREBP target genes, most of which are involved in lipid metabolism, and increase the risk of CAD.

In view of the fact that CAD susceptibility is influenced by many gene polymorphisms and gene-gene interactions. And considering that the lipid regulatory pathway genes FBXW7 and SREBPs are risk factors of CAD among the Uygur Chinese population in our study, we further investigated the effects of the gene-gene interactions on CAD risk. Finally, we found that there is a significant interaction between the recessive model (TT vs. CT + CC) of SREBP-1 and the additive model (CT vs. CC + TT) of SREBP-2; the recessive model of SREBP-1 and recessive model (GG vs. AG + AA) of FBXW7; and the additive model of SREBP-2 and recessive model of FBXW7. In addition, the combination of the three models also exhibited a significant interaction, which suggests that they may have synergistic effects on CAD. The potential mechanism for this interaction is not yet clear, and thus, larger well-designed studies are warranted to validate our finding.

Our results identified a significant association of FBXW7 and SREBP variants with CAD in the Uygur Chinese population but not in the Han Chinese population. On one hand, a possible reason for these differences may be due to the interaction between genetic differences and environmental factors; the Uygur population is a relatively isolated group, accounting for approximately 47% of the total population in Xinjiang, China. Their eating habits and lifestyles are more consistent among their population and are different from those of the Han Chinese population. For example, the Uygur Chinese population primarily ingests high calorie foods, such as pasta, nuts, beef, mutton, and milk products, and exhibits a low intake of vegetables, fruit and rice compared to the Han Chinese population. On the other hand, ethnic differences may also contribute to the different results between the Han Chinese and Uygur Chinese populations.

### Despite the promising findings in this study, several limitations should be mentioned

First, when participants were recruited from our hospital, we did not collect dietary information despite understanding that dietary information may be insightful. Second, the Uygur Chinese population is an admixed population that mainly lives in the Xinjiang Uygur Autonomous Region of China, and there is a lack of individual genetic background information. Third,as there is lack of genome-wide association studies (GWAS) on CAD from which to extrapolate information on rs9902941 of SREBP-1, rs7288536 of SREBP-2 and rs10033601 of FBXW7, additional efforts will be directed to performing GWAS in different populations.

In conclusion, genetic variations rs9902941 of SREBP-1, rs7288536 of SREBP-2 and rs10033601 of FBXW7 are associated with CAD among the Uygur Chinese population in China. However, our results need to be verified by a larger sample sized, multicentre, case-control study. Moreover, functional analyses are also indispensable to provide biological evidence of a causal association.

## MATERIALS AND METHODS

### Ethical approval of the study protocol

We conducted the study in accordance with the Declaration of Helsinki. All participants provided written informed consent of this study protocol. The study was approved by the Ethics Committee of the First Affiliated Hospital of Xinjiang Medical University in Xinjiang, China.

### Subjects

All participants were recruited from the First Affiliated Hospital of Xinjiang Medical University from 2013 to 2016. We enrolled a total of 1,064 CAD patients (Han = 650; Uygur = 414), and the control group included 1,082 participants (Han = 662; Uygur = 420); all participants were unaffected by renal dysfunction, valvular disease and chronic inflammatory disease. Coronary angiography was used to diagnose CAD, which was indicated by the presence of at least one significantly stenotic coronary artery affecting more than 50% of the luminal diameter. Participants of the control group also underwent coronary angiography and were confirmed to be free of coronary artery stenosis. In addition, the participants did not show clinical or electrocardiographic evidence of myocardial infarction (MI) or CAD [38, 39]. However, some of them had cardiovascular risk factors, such as essential hypertension (EH), diabetes mellitus (DM) or hyperlipidemia, but did not have a history of MI or CAD. Information and data regarding EH, DM, hyperlipidemia and smoking status were collected from all study participants, and these parameters were used to match individual CAD patients and controls.

### Biological measurements and the definition of cardiovascular risk factors

Biological parameters, including serum concentrations of TC, TG, FPG, HDL-C and LDL-C, were measured as previously described using standard methods in the Department of Clinical Laboratory at the First Affiliated Hospital of Xinjiang Medical University. Major CAD risk factors were defined based on current national guidelines. Hyperlipidemia was defined as total plasma cholesterol > 6.22 mmol or plasma triglyceride levels > 2.26 mmol and/or the current use of lipid-lowering drugs with an established diagnosis of hyperlipidemia [40]. Hypertension was defined as a mean SBP ≥ 140 mmHg and/or mean DBP ≥ 90 mmHg among 3 measurements or the use of antihypertensive drugs [41]. DM was diagnosed as FPG ≥ 6.99 mmol/L or a prior DM diagnosis and/or the use of a diabetes drug [42]. Smoking status was defined as currently smoking cigarettes.

### DNA extraction

Blood samples were collected from all participants using the standard venipuncture technique and EDTA-containing tubes. As previously described, DNA was extracted from peripheral blood leukocytes using a whole blood genome extraction kit (Beijing Bioteke Corporation, Beijing, China) [43].

### SNP selection

After carefully reviewing the literature, we included the SREBP family (SREBP-1 and SREBP-2) and FBXW7 genes as key genes of the lipid metabolism regulatory pathway [19]. The SREBP-1 gene encodes 1,147 amino acids and is located on chromosome 17p11.2. It contains 21 exons, which are separated by 20 introns. The SREBP-2 gene encodes 1,141 amino acids and is located on chromosome 22q13.2. It contains 23 exons, which are separated by 22 introns. In the present study, we screened the 1000 Genomes (http://www.1000genomes.org/) and Haploview 4.2 software and selected rs9902941 C < T [C is the major allele and T is the minor allele; the information is from dbSNP, National Center for Biotechnology Information (https://www.ncbi.nlm.nih.gov/ projects/SNP/)], which has been identified in previous studies to be a significant variant of SREBP-1 [29]. In addition, we also selected the tag SNP rs7288536 C < T of SREBP-2 (C is the major allele and T is the minor allele; the information is from dbSNP, National Center for Biotechnology Information). The human FBXW7 gene consists of 707 amino acids and is located on chromosome 4q31.3. It contains 17 exons, which are further separated by 16 introns. Similarly, we screened the 1000 Genomes and Haploview 4.2 software and selected rs10033601 A < G of FBXW7 (A is the major allele and G is the minor allele; the information is from dbSNP, National Center for Biotechnology Information). Because there was no reference data for the Uygur Chinese population in the 1000 Genomes (http://www.1000genomes.org/) and Haploview 4.2 software, we obtained the three SNPs by referencing data on the Han Chinese population in Beijing, China (CHB), using a minor allele frequency (MAF) ≥ 0.05 and linkage disequilibrium patterns with *r*^2^ ≥ 0.8 as a cut-off for our analysis.

### Genotyping

SNP genotyping was performed using an improved multiplex ligase detection reaction method (iMLDR, Genesky Bio-Tech Cod., Ltd., Shanghai, China) as previously described [44]. Randomly selected DNA samples from each genotype were sequenced using a ligation detection reaction method to validate the genotyping. The results of the ligation detection reaction analysis were consistent with the results of sequencing.

### Statistical analysis

The mean ± standard deviation (SD) was calculated for continuous variables, and the participants in the CAD and control groups were compared using an independent-sample *t*-test. Categorical variables and the distribution of genotypes and models were shown as numbers and percentages (%), and the two groups were compared using the χ^2^ test or Fisher's exact test. In addition, to compensate for multiple comparisons of genotypes, we applied Bonferroni's correction in the statistical analysis. The Hardy-Weinberg equilibrium (HWE) was evaluated using SNP Stats (available online at http://bioinfo.iconcologia.net/SNPstats). Moreover, logistic regression analysis was performed to assess the contribution of a certain model of variants rs9902941 of SREBP-1, rs7288536 of SREBP-2 and rs10033601 of FBXW7 to CAD, and to CAD with DM. Gene-gene interactions were also evaluated using the multiple logistic regression analysis.The odds ratios and 95% CIs were calculated to determine the strength of the associations between the SNPs and CAD. After adjusting for age; gender; plasma concentrations of TG, TC, HDL-C, LDL-C and FPG; hypertension; and drinking and smoking habits, a multivariate analysis was performed. All statistical analyses were performed using SPSS version 22.0 for Windows (SPSS Inc., USA), and statistical significance was established at an alpha level of 0.05.

## SUPPLEMENTARY MATERIALS FIGURES AND TABLES



## Abbreviations

CAD =: Coronary artery disease
SNPs =: Single-nucleotide polymorphisms
BMI =: Body mass index
FBG =: Fasting blood glucose
TG =: Triglycerides
TC =: Total cholesterol
HDL-C =: High density lipoprotein-cholesterol
LDL-C =: Low density lipoprotein-cholesterol
BUN =: Blood urea nitrogen
Cr =: Creatinine.
