# Supplementary material for: Association of genetic variations in the lipid regulatory pathway genes FBXW7 and SREBPs with coronary artery disease among Han Chinese and Uygur Chinese populations in Xinjiang, China
Source: Oncotarget. 2017 Sep 19;8(50):88199–210. doi: 10.18632/oncotarget.21082 (PMC5675704; doi:10.18632/oncotarget.21082)
Supplement: Supplementary file 1 [file oncotarget-08-88199-s001.pdf]

Association of genetic variations in the lipid regulatory pathway genes FBXW7 and SREBPs with coronary artery disease among Han Chinese and Uygur Chinese populations in Xinjiang, China

SUPPLEMENTARY MATERIALS

Supplementary Table 1: The genotype distribution of selected SNPs in CAD patients with and without diabetes

Variants	CAD with DM *n* (%)	CAD without DM *n* (%)	*P*-value
SREBP-1 rs9902941 C < T			
CC	97 (55.1%)	123 (51.7%)	
CT	65 (36.9%)	89 (37.4%)	0.562
TT	14 (8.0%)	26 (10.9%)	
Dominant model			
CC	97 (55.1%)	123 (51.7%)	0.489
CT + TT	79 (44.9%)	115 (48.3%)	
Recessive model			
TT	14 (8.0%)	26 (10.9%)	0.312
CT + CC	162 (92.0%)	212 (89.1%)	
Additive model			
CT	65 (36.9%)	89 (37.4%)	0.923
CC + TT	111 (63.1%)	149 (62.6%)	
SREBP-2 rs7288536 C < T			
CC	15 (8.5%)	26 (10.9%)	
CT	79 (44.9%)	110 (46.2%)	0.623
TT	82 (46.6%)	102 (42.9%)	
Dominant model			
CC	15 (8.5%)	26 (10.9%)	0.419
CT + TT	161 (91.5%)	212 (89.1%0	
Recessive model			
TT	82 (46.6%)	102 (42.9%)	0.450
CT + CC	94 (53.4%)	136 (57.1%)	
Additive model			
CT	79 (44.9%)	110 (46.2%)	0.788
CC + TT	97 (55.1%)	128 (53.8%)	
FBXW7 rs10033601 A < G			
AA	71 (40.3%)	81 (34.0%)	
AG	72 (40.9%)	113 (47.5%)	0.354
GG	33 (18.8%)	44 (18.5%)	
Dominant model			
AA	71 (40.3%)	81 (34.0%)	0.188
AG + GG	105 (59.7%)	157 (66.0%)	
Recessive model			
GG	33 (18.8%)	44 (18.5%)	0.946
AG + AA	143 (81.3%)	194 (81.5%)	
Additive model			
AG	72 (40.9%)	113 (47.5%)	0.184
AA + GG	104 (59.1%)	125 (52.5%)	

$\chi 2$ test for genotype distributions between coronary artery disease patients and controls
